# Study protocol for the implementation of *Centering Patients with Fibroids*, a novel group education and empowerment program for patients with symptomatic uterine fibroids

**DOI:** 10.1186/s12978-024-01777-2

**Published:** 2024-04-01

**Authors:** Nyia L. Noel, Jasmine Abrams, Estefania Rivera Mudafort, Anagha Babu, Emma Forbes, Lauren Hill, Cherie C. Hill, Tanika Gray Valbrun, Nkem Osian, Lauren A. Wise, Wendy Kuohung

**Affiliations:** 1 Department of Obstetrics & Gynecology, Chobanian and Avedisian School of Medicine, Boston University, Boston Medical Center, Boston, MA USA; 2 Yale School of Public Health, Yale University, New Haven, CT USA; 3 Harvard University, Boston, MA USA; 4 Department of Obstetrics and Gynecology, Emory University School of Medicine, Atlanta, GA USA; 5 The White Dress Project, Atlanta, GA USA; 6 Department of Epidemiology, Boston University School of Public Health, Boston, MA USA

**Keywords:** Uterine fibroids, Education, Advocacy, Patient experiences, Community partnerships, Empowerment, Group model, Centering healthcare institute

## Abstract

**Background:**

Black women and people with uteri have utilized collectivistic and relational practices to improve health outcomes in the face of medical racism and discrimination for decades. However, there remains a need for interventions to improve outcomes of uterine fibroids, a condition that disproportionately impacts Black people with uteri. Leveraging personalized approaches alongside evidence that demonstrates the positive impact of social and peer support on health outcomes, we adapted from *CenteringPregnancy*, an evidence based group prenatal care intervention, for the education and empowerment of patients with uterine fibroids.

**Methods:**

The present report provides  an overview of the study design and planned implementation of CPWF in cohorts at Boston Medical Center and Emory University / Grady Memorial Hospital. After receiving training from the Centering Healthcare Institute (CHI), we adapted the 10-session *CenteringPregnancy* curriculum to an 8-session hybrid group intervention called *Centering Patients with Fibroids* (CPWF). The study began in 2022 with planned recruitment of six cohorts of 10-12 participants at each institution. We will conduct a mixed methods evaluation of the program using validated survey tools and qualitative methods, including focus groups and 1:1 interviews.

**Discussion:**

To date, we have successfully recruited 4 cohorts at Boston Medical Center and are actively implementing BMC Cohort 5 and the first cohort at Emory University / Grady Memorial Hospital. Evaluation of the program is forthcoming.

## Abstract (plain language summary)

Fibroids are non-cancerous smooth muscle tumors that disproportionate impact black women and gender expansive people. Our team adapted *CenteringPregnancy*, a group based model of prenatal care, to an education and empowerment program for peple with fibroids called *Centering Patients with Fibroids (CPWF)*. This paper describes the development and implemation of the program at two academic hospitals serving diverse patients in Boston, Massachusetts and Atlanta, Georgia. To evaluate the successes and challenges of the program, we ask participants to complete surveys to learn more about their experience with having fibroids and also invite them to group feedback sessions or focus groups. We also interview other healthcare providers, team members, and hospital leadership on their knowledge and thoughts about the program. We hope to use the feedback to improve the program and make it available to more people across the country.

## Background

Despite major advances over the past several decades, the medical field has made minimal progress in addressing health disparities, especially in maternal and reproductive health. The Black Women’s Health Imperative, an organization founded to address health disparities and reproductive rights of Black women, recently celebrated their 40 year anniversary [1]. The organization’s premier event was the first National Conference on Black Women’s Health Issues held at Spelman College in Atlanta, Georgia in 1983. In a letter addressed to conference attendees, founder and reproductive health leader Byllye Y. Avery raised a call to action: “The conception of this project and the birth of this Conference are no coincidence.…Our presence at this "conference of the decade" indicates our commitment to improving the health of Black women and our support of the Women’s Health Movement…The time to rise to action is NOW!” [2]. Avery’s conference of nearly 2,000 attendees, the coming together of “self-help group” members from around the country, reframed racism in women’s health in the public, medical, and academic spheres, and provided group space for Black women to share experiences and collaborate towards systemic solutions. The 3-day conference was a pivotal event in the history of reproductive health and rights advocacy, and 40 years later, Black women continue to experience disproportionately negative health outcomes on nearly every indicator of reproductive health. Thus, community organizing and novel interventions for Black reproductive health remain relevant and needed today.

Uterine fibroids (UF), non-cancerous smooth muscle tumors of the uterus, are the most common tumor in people with uteri and disproportionately impact Black people and those of African ancestry [3–10]. Black people with UF report feeling dismissed [11], undervalued [11], and encouraged to seek hysterectomy when they would prefer uterine and/or fertility-sparing management options [12]. Implicit racial bias within the healthcare system has been directly linked to delivery of lower quality care for Black patients [13]. Such practices, identified as medical racism, affect Black patients’ decisions to seek care for symptomatic UF, provider perceptions of patient pain and symptoms, and treatment options offered for UF management.

In the field of Obstetrics and Gynecology, *CenteringPregnancy* is lauded as a standout intervention to improve maternal and neonatal outcomes. *CenteringPregnanc*y combines group education with individual directed prenatal care for pregnant patients with a similar estimated due date. *CenteringPregnancy* group sessions include pregnant patients and providers discussing nutrition, stress management, labor and delivery, breastfeeding, and infant care in addition to routine aspects of the clinical visit. Participation in *CenteringPregnancy* has led to decreased rates of preterm and low birth weight infants, an increase in breastfeeding/chestfeeding rates, and increased spacing between pregnancies [14].

Another key aspect of the intervention is our partnerships with community and national advocacy groups, The White Dress Project (TWDP) and the Boston-based Resilient Sisterhood Project (RSP). The White Dress Project (TWDP) is a national nonprofit patient advocacy organization dedicated to raising global UF awareness through education, research, and community-building. Since its founding in 2014, TWDP has established the month of July as Fibroid Awareness Month in the US House of Representatives, lobbied successfully for the Stephanie Tubbs Jones Fibroid Research and Education Act, and curated educational webinars, regional events, and a robust social media presence. RSP is a Boston-based nonprofit that supports the gynecological health of women of African descent whose members and executive director assisted in the first cohort of CPWF.

Group-based models of care contribute to the development of a network of patients, providers, and peer advocates who work together to prioritize patients and improve health outcomes, creating spaces ripe for promoting health education and literacy while inspiring patient autonomy. Our team adapted the *CenteringPregnancy* model to develop and implement a group education model, *Centering Patients with Fibroids* (CPWF), an intervention that we hypothesize will improve knowledge, self-advocacy, reproductive health outcomes, and quality of life for black patients with UF.

## Methods/design

This pilot prospective cohort trial will utilize a concurrent mixed-methods design to obtain information about intervention feasibility, acceptability, and sustainability and assess preliminary evidence of improved outcomes.

### Intervention

We will implement *Centering Patients with Fibroids* (CPWF) at Boston Medical Center (BMC) and Grady Memorial Hospital/Emory University Hospital Midtown, two academic-based safety net hospitals that serve racially and socioeconomically diverse patient populations in different regions of the United States. The investigation of patients at these two hospitals will help to detect any existing regional differences in implemention. The study was approved by the Institutional Review Board at both institutions. Eligible participants include patients between the ages of 18 to 45 years old receiving care at one of the study hospitals, with symptomatic UF measuring equal or more than 2 cm, and uterine dimensions of less than 20 cm on pelvic imaging within 12 months of recruitment. Exclusion criteria include inability to speak English, pregnancy at time of recruitment, and no available pelvic imaging. Eligible and interested patients will provide informed consent to participate in an 8-week education and empowerment program, conducted in person and via Zoom in a hybrid modality. Each 1.5 h session will be facilitated by the study site principal investigator (PI), research staff trained in group facilitation by the Centering Healthcare Institute (CHI), and/or members of our advocacy partner, The White Dress Project.

**Fig. 1:**
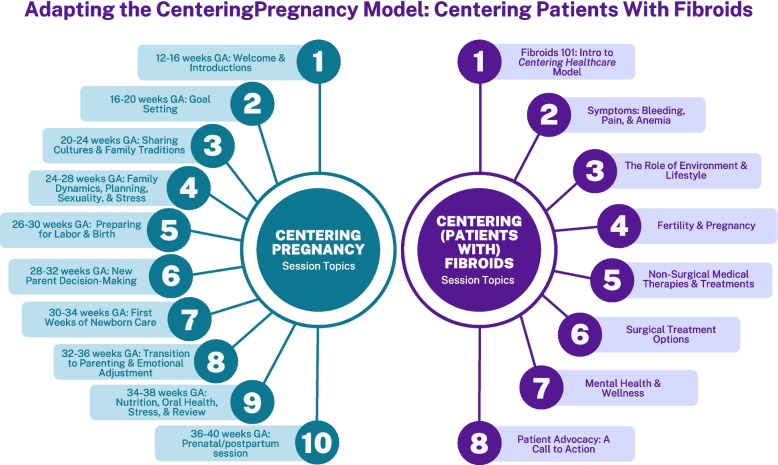
Adapting the CenteringPregnancy model for uterine fibroids

Our investigative team adapted the components of the *CenteringPregnancy* program, a group model of prenatal care developed through the Centering Healthcare Institute (CHI), to design a complementary education and empowerment program for patients with UF. CHI’s group care framework prioritizes high quality healthcare, interactive learning, and positive health outcomes through building relationships and community among group members. To apply and adapt this model to serve the needs of patients with UF, our research team members were first trained as certified Centering facilitators. CHI facilitator training provides a broad range of strategies for both virtual and in-person engagement, including mindfulness activities, storytelling exchanges, writing exercises, and “ball-toss” interactive conversations. Storytelling exchanges in particular can help to empower and improve patient engagement in their own care by sharing personal experiences [15, 16]. In the broader context, storytelling also has the power to reduce health disparities [17]. Our team used the skills gained from this facilitation training to create CPWF as a group-based care model focused on providing exceptional care to patients with UF through a multidisciplinary, patient-centered, conversational, and evidence-based approach. CPWF curriculum topics mirror *CenteringPregnancy* and include the role of environment and diet on UF; impact of UF on mental health, fertility and pregnancy, bleeding and anemia, and pain; and UF treatments including non-surgical medical therapies, uterine-sparing procedures, and hysterectomy (Fig. 1). Program participants receive detailed information on all of the surgical and non-surgical options for managing UF and have the chance to discuss their options with peers and clinical staff.

Healthcare advocates from TWDP co-facilitate each session to aid in maintaining a patient-centered experience.

CPWF cohorts are implemented using a hybrid modality, with the first and last sessions conducted in person, and the remaining sessions conducted via Zoom (Fig. 1). A total of 7 cohorts will receive the program at BMC, and a total of 6 cohorts will receive the program at Emory/Grady. All CPWF participants complete a “Dear Fibroids” letter to their fibroids at the beginning of the session, a personal effort to define and process how fibroids have impacted their daily lives. We utilize Zoom features such as breakout rooms, screen sharing, and polls for greater engagement in the virtual space. For example, one strategy for online engagement includes activities to establish rapport between patients via Zoom breakout rooms, focused on discussing fibroid experiences, unique or fun facts about participants, and hobbies. Other strategies include mindfulness activities, “ball toss” activities to reinforce learning in a playful way, and artistic exercises such as drawing in the “Past, Present and Future” exercise in which participants color a tree with branches symbolizing various stages in their fibroid journey.

### Recruitment

**Fig. 2:**
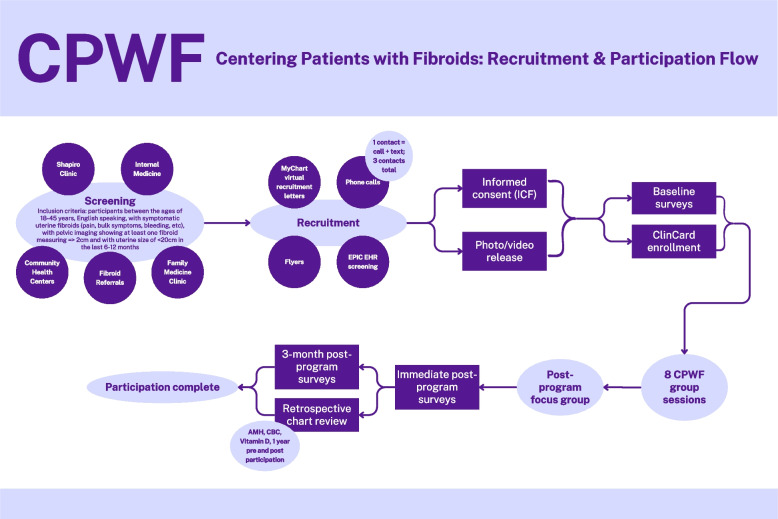
Centering Patients with Fibroids (CPWF) recruitment and participation flow

Participants will be recruited from the Fibroid Center and Obstetrics and Gynecology (OBGYN) clinics at Boston Medical Center, Emory University Hospital Midtown, and Grady Memorial Hospital. Participants will be identified using the BMC, Emory University Hospital Midtown and Grady Memorial Hospital Electronic Medical Records (EMR), Epic MyChart, clinic schedule, and flyers placed in high-traffic areas in the clinical space (Fig. 2). Potential participants may also be referred from OBGYN providers in the clinic if they meet criteria. A research team member reaches out to patients who meet inclusion criteria by phone or during an in-person clinic encounter to inquire about interest in participating in the study. If a candidate for the study agrees to participate, a trained study researcher/interviewer will contact the subject and explain what the study entails to obtain informed consent.

In addition to these screening and recruitment practices, we used EPIC/MyChart to reach patients not seen in the OB/GYN practice who are diagnosed with UF. We obtained a list of non-pregnant patients with fibroid diagnoses as a preliminary eligibility screening, then sent a study recruitment letter with Opt Out language to the patient’s MyChart inbox. We posted digital flyers with QR codes on waiting room television monitors in clinics throughout the hospital to supplement paper recruitment flyers. We reached out to the Family Medicine and General Internal Medicine departments at BMC and Emory/Grady to identify provider research champions to assist us in identifying potentially eligible patients/connecting them with the study opportunity through MyChart. Finally, we will access emergency department and gynecology follow up referral lists to screen patients for CPWF and reach out to potentially eligible subjects. Participants will be remunerated up to $200 via reloadable gift cards called “ClinCards” for survey completion, lab draw, and session attendance. We emphasize that CPWF is a program designed to complement, rather than replace, one-to-one gynecological care with a participant’s provider.

In addition to recruiting patients, we recruited healthcare leaders and providers at Boston Medical Center, Emory University Hospital Midtown, and Grady Memorial Hospital to participate in interviews. We will identify those who are invested in improving access to quality care and health outcomes for patients seeking care at BMC, Emory University Hospital Midtown, and Grady Memorial Hospital. Interviewees will be leaders and providers who have had some level of contact with the Fibroid Center, Emory University Hospital Midtown, and Grady Memorial Hospital or provide care to women seeking care related to symptomatic uterine fibroids. They are contacted via email and asked to participate. If they agree, we will obtain consent and schedule interviews.

### Quantitative methods

To assess preliminary program impact on physical and mental health outcomes, participants will complete self-reported surveys during their participation in this study: pretest, posttest, and 3 month follow up. The surveys will include demographic questions and a series of validated measures, including the Uterine Fibroid Symptom and Quality of Life (UFS-QOL), the Everyday Discrimination Scale, Patient Health Questionnaire-9 (PHQ-9), and the Instrument for Evaluation of the Experience of Chronic Patients (IEXPAC).

As the aim of this pilot study is to assess preliminary outcomes as well as feasibility, acceptability, and sustainability, achieving a predetermined sample size for power was not necessary. We anticipate 10-12 participants per cohort for a total of 13 cohorts, 7 at BMC and 6 at Emory/Grady. As such, we will perform comparison of mean scores of the various validated survey instruments using analysis of variance (ANOVA) tests. We will also ask participants to give blood for serum studies including complete blood count (CBC), Anti-Müllerian hormone (AMH), and 25-OH-Vitamin D. Values will be compared at baseline and 3 months after the program completion. We hypothesize decreased anemia and vitamin D deficiency among program participants at 3 month follow up, and variable change in the AMH level related to the age at the time of program participation.

### Survey measures

#### Uterine Fibroid Symptom and Quality of Life (UFS-QOL)

The UFS-QOL is designed to assess symptomology and health-related quality of life for patients managing UF. Scores are calculated on a scale of 0-100, with higher scores indicating a higher reported quality of life [18]. The first 11 questions are framed using a Likert scale (Never; Almost Never; Sometimes; Almost Always; Always) and score from 0-10, with higher scores indicating a better patient experience in the past 6 months. The final 3 questions, reported separately, describe continuity of healthcare after hospitalization, emergency department care, home health care, and social services utilization [19].

#### Everyday discrimination scale

The Everyday Discrimination Scale measures experiences of discrimination in day-to-day life based on racial identity, ancestry and tribal identity, gender and sexuality, income and education, age, religion, ability, and physical appearance including height, weight, and skin tone. Scores range from 0–25, with higher scores indicating more experiences of discrimination [20–22].

#### Patient Health Questionnaire-9 (PHQ-9)

The PHQ-9 screens, diagnoses, and assesses symptom severity for depression over the past 2 weeks on a 4-point Likert scale (Not at all; Several days; More than half the days; Nearly every day), with final scores ranging from 0-27. Major depressive disorder is suggested if 5 or more items are marked as “More than half the days,” and if item 1 or 2 is marked as at least “More than half the days.” Other depressive syndrome is suggested if 2-4 items are marked as at least “More than half the days,” and if item 1 or 2 is checked as at least “More than half the days.” Participants are considered to screen positive for depression if their overall score is 5 or higher [23, 24].

#### Instrument for Evaluation of the Experience of Chronic Patients (IEXPAC)

The IEXPAC survey was developed in the Chronic Care Model theoretical framework [25] and scores participants’ replies to questions about their experience with integrated care for chronic health conditions. IEXPAC focuses on patients’ experiences with entire care teams, and aligns with the Triple Aim framework of improving individual healthcare experience, improving population health, and reducing per capita healthcare expenditures [26]. Overall scores range from 0 (worst experience) to 10 (best experience) [26, 27]. IEXPAC was developed and validated in 2016 to assess patient self-reported experience in the chronic illness context.

### Qualitative methods

#### Methodology and theoretical framework

The qualitative component of this study utilizes methodological triangulation of data: (1) interviews with providers, (2) interviews with patients, and (3) process mapping. Collectively, this formative research will generate comprehensive data needed for improved intervention implementation, scalability, and sustainability.

The Practical, Robust Implementation and Sustainability Model (PRISM) helps facilitate the translation of research into practice by providing a framework for understanding how to promote optimal effectiveness of health services interventions, given the influence of multilevel factors that impact intervention implementation and patient outcomes [22]. PRISM asserts that success of intervention implementation depends on complex interactions between programs, key players, and environments. Specifically, the model details how healthcare interventions influence and are influenced by organizational and patient level factors and how these interactions ultimately impact intervention adoption, implementation, maintenance, reach, and effectiveness (Fig. 1 - Prism Framework) [22]. Based on the PRISM framework, which values and incorporates patient and provider experiences and perspectives, process mapping will be used to comprehensively describe in detail step-by-step patient and provider journeys for CPWF.

#### Individual interviews

Conducting interviews with both patients and providers will be essential for understanding the experiences of each group, which will likely have different perspectives and unique characteristics that will be important to consider for intervention adaptation, implementation, scalability, and sustainability. As such, interviews will be conducted to clarify (a) how patients enroll in CPWF, receive fibroid diagnoses, and engage in educational content and programming regarding treatment options; (b) organizational, cultural, and behavioral barriers and facilitators of engagement in screening and treatment; and (c) areas of possible implementation failure of the education program.

#### Recruitment

Convenience and purposive sampling strategies will be used to recruit 15 patients and 15 providers from each hospital for qualitative interviews. Based on previous work and standards in qualitative research, 15 interviews per demographic is sufficient to obtain thematic data saturation. Additional interviews will be conducted if needed to reach saturation. Eligible providers work at partnering hospitals and actively provide care for patients with UF. Providers will be recruited via email, word of mouth, and participant referral.

**Fig. 3:**
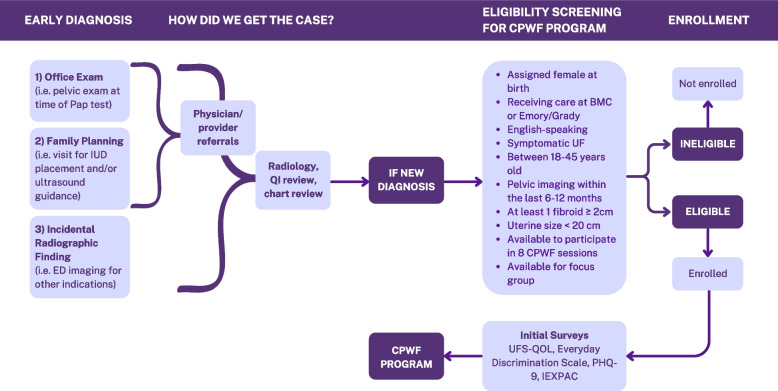
Programmatic flow diagram

Participants in CPWF at partnering hospitals ages 18 to 45, with a new diagnosis of UF, will be eligible to participate. We will recruit patients with UF discovered at the time of: routine office pelvic examinations, exams in the setting of intrauterine device placement requiring bimanual exam and/or ultrasound guidance, and audits of pelvic imaging within the last 12 months done at the study institutions with incidental findings of uterine fibroids. Included participants had pelvic imaging showing at least one fibroid measuring equal or more than two cm and with uterine size less than twenty cm within the last six to twelve months. Targeted recruitment will be conducted through flyers emailed to providers and posted in clinical areas. Self-referral mechanisms are also in place in addition to patient recruitment in clinical waiting rooms and through clinic EHR schedule reviews. Providers who newlydiagnose UF will also refer patients to the study (Fig. 3).

#### Procedure

All interviews will be conducted in English by a health psychologist with 15 years of expertise in qualitative research and trained research assistants. Informed by the PRISM model, the interview guide will be divided into three sections to reflect data collection goals. Interviews will be recorded, transcribed, verified, and entered into NVivo12, a qualitative data analysis software program, for analysis. Information from the interviews will be used to inform the: (1) process mapping, (2) tailoring of intervention content to address patient and organization specific factors that impact engagement in and implementation of the educational program, (3) creation of activities that directly incorporate aspects of culture to promote engagement in program, and (4) inclusion of culture-specific terminology in materials.

#### Qualitative data analysis

Thematic analysis will be conducted and guided by a five step process outlined by Braun and Clarke to identify key themes that correspond to the theoretical model, PRISM.21 The steps are: (1) Become familiar with the data by reviewing all transcripts, (2) Generate initial codes, (3) Identify themes, (4) Review/revise themes, and (5) Define and name themes. Trained research assistants (RAs) will review each of the transcripts to develop a preliminary coding scheme.

Using NVivo 12, the RAs will code all of the data independently using the preliminary coding scheme as a guide and will create new codes as needed. The RAs will then meet to discuss any coding disagreement until a minimum 90% agreement is achieved. Data analysis will be cyclical and involve continuous development of new codes and constant comparison of themes. To reduce bias, enumeration will be utilized. That is, the frequency of codes applied to transcribed data will be tallied and prevalent codes will be used to form initial thematic categories.

#### Process mapping

Process mapping is a useful strategy for characterizing and evaluating healthcare processes such as patient navigation. Utilization of process maps facilitate ease of understanding complex processes through visual illustrations. Data to complete the map will be gathered via observation and interviews to clarify processes and timelines. Upon determining steps in the process map and associated timelines, a visual representation of the process will be developed. Next, the research team will identify areas of potential failure to implement the intervention using the process map and the possible causes and consequences of potential implementation failures. The goal is to identify optimal timing for implementation of the CPWF intervention components, areas of potential intervention failure, and strategies to help avoid implementation failure.

## Discussion

The Centering Healthcare Institute (CHI) was founded in 2001 by nurse-midwife Sharon Rising, MSN, CNM, FACNM to transform and expand care for pregnant patients [28]. The CHI model aims to adapt patients’ routine clinical health assessment to the group setting, provide interactive adult learning experiences on health diagnosis and treatment options, and build community among individuals with shared and similar healthcare experiences. *CenteringPregnancy,* the first program to be implemented by CHI, has been shown to improve patients’ and providers’ experiences of care, achieving better care quality, improved health outcomes, and lower costs. When compared with patients who received traditional prenatal care, *CenteringPregnancy* participants experienced 33-47% lower rates of preterm birth across published studies. These reductions in preterm birth rates were especially high for birthing parents of African American and Latinx identities [14]. Participation in *CenteringPregnancy* also leads to dramatic increases in breastfeeding/chestfeeding initiation and continuation, reduction in incidence of sexually transmitted infections in pregnant adolescent patients, lengthening of the key interconceptional period between pregnancies in pregnant adolescent patients, and higher patient satisfaction with the healthcare system [14].

The *CenteringPregnancy* model has been adapted to improve women’s health, and efforts to explore group care as a pathway to gynecological health equity continue to grow nationally. CPWF pilots the way in which chronic gynecological conditions can be treated within the group care framework by applying Centering approaches to UF, a common condition with intense racial disparities in care [29]. CPWF is the first program of its kind to adapt the CHI group care framework to gynecological practice. Like CenteringPregnancy, we anticipate that CPWF will reduce healthcare costs and improve patient and provider care experiences, care quality, health outcomes.

Our study group will describe the experiences of participants and feedback from providers and other healthcare leadership on program implementation and will identify modifiable intervention targets and organizational, provider, and patient level factors associated with successful implementation of CPWF. Quantitative data include survey results and serial lab values for hemoglobin, hematocrit, vitamin D, iron studies, and AMH. Hemoglobin and hematocrit are markers of anemia, which is prevalent in patients with heavy menstrual bleeding due to UF [30]. Vitamin D deficiency is a possible risk factor for UF occurrence. Vitamin D concentrations have been found to be inversely associated with uterine fibroid prevalence, especially in Black women [31]. AMH is used as a marker of functional ovarian reserve [32] and is thus valuable in counseling women about reproductive and treatment planning in the setting of gynecological conditions such as UF [33]. The combination of both qualitative and quantitative methods will allow us to capture the benefits of CPWF over a shorter timeframe and to lay the groundwork for larger studies. We will use the data from our mixed methods study to direct next steps, including future iterations of the curriculum in response to feedback from participants, faculty, clinicians, and other key stakeholders, and a larger multisite randomized controlled trial to further explore the efficacy and generalizability of CPWF across the country.

Our findings may demonstrate the feasibility of applying the Centering approach to other gynecologic disease entities impacted by racial disparities such as endometriosis. Though our work is centered around the patient experience and health outcomes, sustainability must include a business case to demonstrate return on investment in the program for health systems and hospitals through cost savings or revenue generation. Program uptake could change referral patterns and health care utilization by patients opting for this model of education to complement their relationship with a gynecologist. We anticipate that the sharing of patient experiences with uterine fibroids, built community and engagement with clinicians, subject matter experts, and national health advocates will continue the tradition of Black women building resilience and promoting health through collectivistic practices such as that observed at the 1st National Conference on Black Women’s Health in 1983. *Centering Patients with Fibroids* has the potential to reduce health disparities in patients with UF by improving education, and empowering patients to advocate for themselves in the healthcare setting.

## Data Availability

No datasets were generated or analysed during the current study.

## Abbreviations

TWDP: The White Dress Project
CPWF: Centering Patients with Fibroids
UF: Uterine Fibroids
BMC: Boston Medical Center
CHI: Centering Healthcare Institute
RSP: The Resilient Sisterhood Project
PRISM: Practical, Robust Implementation and Sustainability Model
